# The Theory Behind the Age-Related Positivity Effect

**DOI:** 10.3389/fpsyg.2012.00339

**Published:** 2012-09-27

**Authors:** Andrew E. Reed, Laura L. Carstensen

**Affiliations:** ^1^Department of Psychology, Stanford UniversityStanford, CA, USA

**Keywords:** positivity effect, aging, emotion regulation, motivation, attention, memory

## Abstract

The “positivity effect” refers to an age-related trend that favors positive over negative stimuli in cognitive processing. Relative to their younger counterparts, older people attend to and remember more positive than negative information. Since the effect was initially identified and the conceptual basis articulated (Mather and Carstensen, 2005) scores of independent replications and related findings have appeared in the literature. Over the same period, a number of investigations have failed to observe age differences in the cognitive processing of emotional material. When findings are considered in theoretical context, a reliable pattern of evidence emerges that helps to refine conceptual tenets. In this article we articulate the operational definition and theoretical foundations of the positivity effect and review the empirical evidence based on studies of visual attention, memory, decision making, and neural activation. We conclude with a discussion of future research directions with emphasis on the conditions where a focus on positive information may benefit and/or impair cognitive performance in older people.

## The Theory Behind the Age-Related Positivity Effect

The positivity effect refers to a relative preference in older adults (compared to younger adults) for positive over negative material in cognitive processing. Since the first explicit reference to the positivity effect in 2004 (Kennedy et al., 2004) more than 100 peer-reviewed articles have addressed the concept^1^. This flurry of scholarship has provided overwhelming support for the basic concept but also has added to our understanding of the subtleties and limitations of the theory while enriching our understanding of the role of emotion in cognitive processing in both younger and older people. Below we articulate the operational definition of the positivity effect and ground it in the theoretical framework of socioemotional selectivity theory (SST; Carstensen, 2006). We then consider the empirical literature, arguing that the pattern of findings that has emerged in recent years better supports a top-down, motivational explanation for positivity than accounts that attribute positivity to cognitive or neurological decline. Finally, we propose potential future research directions and discuss ways in which the positivity effect may exert beneficial and detrimental influences on cognitive processing.

## The Operational Definition and Theoretical Foundation of the Positivity Effect

Our research group coined the term “positivity effect” to describe mounting evidence that older adults show a relative preference for positive over negative information in attention and memory (Charles et al., 2003; Mather and Carstensen, 2003; Mikels et al., 2005). “Effect” was chosen over “bias” when the term was coined because age differences are as frequently driven by a preference for negative material in the young as they are driven by a preference for positive material in the old. The positivity effect concerns the relative difference between older and younger people in attention to and memory for *positive* as opposed to *negative* material^2^.

The positivity effect was initially identified by investigating postulates of SST, a life-span theory of motivation (Carstensen, 1993, 2006; Carstensen et al., 1999). According to SST, a core constellation of goals operates throughout adulthood, including basic goals associated with attachment and control as well as goals associated with instrumental needs and emotional gratification. The key postulate of SST is that the relative importance of goals within this constellation changes as a function of future time horizons. Because chronological age is inversely associated with actual and perceived time left in life, systematic age differences emerge in preferred goals. Importantly, according to SST, age differences in goal hierarchies reflect perceived future time more than time since birth (viz., chronological age). When the future is perceived as long and nebulous, as it typically is in youth, future-oriented goals related to gathering information and expanding horizons are prioritized over emotional gratification. When time horizons are constrained present-oriented goals related to emotional satisfaction and meaning are prioritized over goals associated with long-term rewards. In addition to emphasizing changes in goals with age, the theory predicts that when younger people perceive time constraints or older people perceive the future as relatively long, age differences are reduced or eliminated. A number of empirical investigations have supported this claim (e.g., Fredrickson and Carstensen, 1990; Fung et al., 1999; Fung and Carstensen, 2004). When life’s fragility is made salient by events like September 11th or the SARS epidemic in Hong Kong, for example, age differences in socioemotional goals disappear (Fung and Carstensen, 2006). Similarly, under experimental conditions that extend time horizons, older peoples’ goals closely resemble younger peoples’ goals (Fung et al., 1999). Thus, the influence of time horizons on goals has been well-established. The theoretical perspective of SST argues that age-related changes in goals are adaptive, reflecting the reality that changing time horizons and ultimately mortality impose. SST incorporates an evolutionary component that presumes considerable advantages to life course changes in goals. Focusing on individual strivings early in life and focusing on emotional goals later in life, which typically benefit kin, improves reproductive success (see Carstensen and Löckenhoff, 2003). The presence of grandparents increases the survival odds of grandchild offspring in humans and some other mammals, for example (Hawkes, 2003).

Socioemotional selectivity theory maintains that perceived time horizons play an important role in signaling these shifts in motivation. When futures are long and nebulous, acquiring knowledge and exploring help prepare individuals for an array of uncertain challenges looming ahead. As time horizons grow shorter, future-oriented goals related to preparation for the long-term grow less important and present-oriented goals related to emotional meaning, emotion regulation, and well-being gain in priority. Accordingly, many observed age-related changes in emotion, cognition, and behavior are presumed to be top-down and fluid (varying as a function of motivation) rather than bottom-up and fixed (varying as a function of biological aging or experience).

Early in the last decade, our research team began to test hypotheses about the ways in which motivational changes postulated by SST may influence cognitive processing. These efforts expanded upon a large and rich literature in psychology documenting the powerful influence that goals exert on cognitive processing. From classic studies by Neisser and colleagues on inattentional blindness (e.g., Neisser, 1979) to more recent studies on the subconscious priming of explicit goals (e.g., Chartrand and Bargh, 1996; Moskowitz, 2002), the literature has revealed powerful top-down effects of goals on information processing. We reasoned that because chronically activated goals appear to change systematically with age, such changes may consequently direct attention and memory toward or away from emotional material in systematic ways.

When our research team began to examine questions about potential effects of motivation on cognitive processing, previous findings suggested that whereas younger people appear to privilege negative information in cognitive processing (Baumeister et al., 2001; Rozin and Royzman, 2001), older people commonly privilege positive information. Indeed, several early studies found a classic crossover interaction between age and valence (e.g., Mather et al., 2004; Mikels et al., 2005). Of course, a positive processing preference can result from heightened processing of positive *and/or* reduced processing of negative information. Even when older adults show greater attention to negative than positive but attend significantly less to negative than younger adults, the pattern would qualify conceptually as a positivity effect.

Accumulating evidence indicates that the positivity effect emerges reliably from all combinations of heightened processing of positive and reduced processing of negative information. In some studies, age differences are driven by younger peoples’ greater attention to and/or memory for negative material (e.g., study 2 in Charles et al., 2003; study 2 in Ready et al., 2007; Shamaskin et al., 2010). In other studies differences reflect relatively deeper processing of positive material by older people (e.g., Isaacowitz et al., 2006b; study 3 in Mather and Knight, 2005). Several investigations find that while both older people and younger people attend to negative stimuli more than positive, older people do so significantly less than younger people (e.g., Comblain et al., 2004; Kensinger et al., 2007).

The positivity effect has been documented across a variety of experimental paradigms and a wide range of stimuli, also supporting the robustness of the effect. Studies of visual attention using dot-probe and eye-tracking paradigms show that, compared to younger adults, older adults direct their gaze toward happy and away from angry or sad faces (Mather and Carstensen, 2003; Isaacowitz et al., 2006a,b). The positivity effect also emerges in studies of working memory (Mikels et al., 2005), short-term memory (Charles et al., 2003), autobiographical memory (Kennedy et al., 2004; Schlagman et al., 2006), and even false memories (Fernandes et al., 2008). Compared to younger adults, older adults appear to privilege positive over negative stimuli across a wide range of experimental materials including emotionally valenced images (Charles et al., 2003; Spaniol et al., 2008), word lists (Piguet et al., 2008), emotional faces (Mather and Carstensen, 2003; Leigland et al., 2004), and health-related messages (Shamaskin et al., 2010). Such findings suggest that the effect is not limited narrowly to a certain type of stimuli. The positivity effect is also evident in decision making. Compared to younger people, older people pay greater attention to positive as compared to negative attributes when choosing among doctors and hospitals (Löckenhoff and Carstensen, 2007, 2008), cars (Mather et al., 2005), and consumer products (Kim et al., 2008). Compared to younger adults, older adults also remember their choices in a manner that is positively skewed – either via disproportionately recalling positive attributes and/or via attributing positive attributes to chosen options and negative attributes to rejected options (Mather and Johnson, 2000; Mather et al., 2005; Löckenhoff and Carstensen, 2007, 2008).

Although the majority of empirical findings have been interpreted through the lens of SST, viable alternative explanations for the empirical phenomenon have been offered. Most notably, Labouvie-Vief et al. (2010) have argued that positive material is preferred by older people because negative information, by comparison, is more cognitively demanding. Along similar lines, the aging-brain model proposed by Cacioppo et al. (2011) suggests that the positivity effect in memory^3^ arises from age-related neural degeneration in the amygdala leading to dampened emotional responses to negative stimuli. In addition to these theoretical alternatives, several investigators have failed to observe age-related positivity effects. The literature is now sufficiently large and the methodologies sufficiently diverse that conceptual alternatives and empirical findings – especially those that have failed to observe positivity – can be examined in ways that clarify the nature and source of these intriguing age differences.

The motivational perspective of SST provides clear and testable predictions about the conditions under which the positivity effect should appear and when it should not. As noted above, positivity theoretically reflects controlled cognition, is driven by chronically activated goals, and is adaptive for well-being. Thus, the effect should be most evident when individuals have sufficient cognitive resources to direct attention, when processing occurs under the scope of conscious (as opposed to automatic) control, when individuals are allowed to pursue chronically activated goals without external interference, and when regulating emotions contributes to well-being. Conversely, the effect should not appear when cognitive resources are limited, when information processing is automatic, when contexts impose situation-specific goals that conflict with chronically activated goals, and when prioritizing emotion regulation has significant risks. To assess the empirical support for these predictions we review the extensive research literature on the positivity effect.

In reviewing the empirical literature we focus on three key conceptual issues: First, we present evidence suggesting that the positivity effect represents controlled processing not cognitive decline. Second, we critically examine experimental procedures and conditions under which the positivity effect is and is not observed, illustrating that the effect is malleable rather than unreliable. Finally, we consider whether effect is adaptive or maladaptive for older adults’ everyday functioning.

## Controlled Processing or Cognitive Decline?

According to the motivational perspective offered by SST, the positivity effect stems from age-related shifts in goal priorities that increase the salience of emotionally gratifying information in attention and memory. Because cognitive resources are required to direct information processing toward goal-relevant stimuli and away from less relevant stimuli (Mather, 2006), the positivity effect will be most evident in individuals with relatively good cognitive control. This postulate clearly distinguishes SST from explanations rooted in cognitive decline. Reasoning from the latter positions, if positive material were preferred because negative material is difficult to process, individuals low in cognitive control would show the strongest preference for positive material. Mather and Knight (2005) conducted two studies to examine the role of cognitive control in positivity. In the first study, older and younger participants were asked to view a series of emotionally evocative and neutral pictures. After a 20-min delay, participants were administered an incidental recall test. Compared to younger participants, older participants recalled a greater proportion of positive images and a lesser proportion of negative images. In subsequent analyses, the researchers examined individual differences as a function of cognitive control and found that the positivity effect was most evident in participants with high levels of cognitive control. In a subsequent study, older and younger participants were asked to view the same images from the first study while they monitored and detected changes in a sequence of sounds. In this dual-task paradigm, older adults recalled a greater proportion of negative images and fewer positive images relative to younger adults. In other words, when cognitive resources were experimentally diverted, the preference for positive over negative information was reversed. Using the same divided-attention task, Knight et al. (2007) found similar effects in visual attention. When attention was divided (viz., participants performed a tone-detection task while viewing experimental materials), older adults spent more time than younger adults viewing negative than positive pictures and faces. In contrast, when asked to simply to view the images, older adults attended more to positive versus negative stimuli. Younger adults showed the opposite pattern. These findings indicate that positivity effects depend on the availability of cognitive resources. Positivity is evident when resources are relatively abundant and undivided, but absent when resources are relatively meager or divided.

The critical role of cognitive resources in the positivity effect is further highlighted by research comparing the emotional memory of healthy younger and older adults with older adults suffering from Alzheimer’s disease (AD; Fleming et al., 2003). When all three groups were asked to recall lists of positive, negative, and neutral words, AD patients remembered a greater proportion of negative versus positive words compared to both control groups^4^. In combination with findings from Mather and Knight (2005) the observed patterns essentially rule out cognitive decline as a root cause of positivity. Not only do people who are low in cognitive reserves show the least positivity, they sometimes favor negative information.

Explanations for the positivity effect based on motivation versus degradation are distinguished not only by their emphasis on cognitive resources, but also by predictions regarding the automaticity and temporal signature of positivity. Cognitive decline and neural degradation-based accounts are premised on assumptions that positivity arises from automatic processes associated with affect optimizing (Wurm, 2011) or amygdala dysfunction (Cacioppo et al., 2011), respectively. By contrast a motivational account attributes positivity to more controlled shifts in attentional resources. Thus, the time course of attentional preferences is important. Automatic accounts would predict immediate evidence of positivity whereas SST predicts a somewhat delayed onset. Existing findings support the latter perspective. The time course of attentional preferences for pairs of faces (in which one is emotional and the other is neutral) indicates a delayed onset of positivity consistent with a deliberate re-allocation of resources (Isaacowitz et al., 2009a). In the latter study, which used eye-tracking to discern a precise timeline of gaze patterns, older adults’ preferential attention toward positive stimuli emerged relatively late after stimulus presentation (500 ms). Attentional diversion from negative faces was slower still (3 s). Whereas an automatic account of the positivity effect would be associated with rapid onset of selective attention, findings suggest that older adults’ early attention – i.e., within 500 ms of stimulus onset – is actually skewed *away* from positive faces, and that their fixation biases toward positive and away from negative faces increases over time. Complementary evidence for the delayed onset of positivity was reported by Williams et al. (2006). They used an event-related potential (ERP) paradigm to track the temporal pattern of neural responses while people viewed emotional faces. As in the study by Isaacowitz et al. (2009a), Williams et al. did not observe a positivity effect in the rapid processing of emotionally salient stimuli. On the contrary, age was associated with reduced activation in the medial prefrontal cortex within 150 ms of viewing happy faces. Yet activation increased later (180–450 ms after onset) in processing of fearful faces. This pattern suggests that only responses to fearful faces are down-regulated.

The lack of positivity for relatively automatic processing is also evident in memory for arousing versus non-arousing words (Kensinger, 2008). Kensinger presented lists of words varying systematically in both valence and arousal and subsequently tested incidental memory. Although a positivity effect was observed in memory for non-arousing emotionally valenced words, older and younger adults showed equivalent recall for arousing positive and negative words, which appear to be processed in a more automatic manner than non-arousing words (for a discussion, see Kensinger, 2004). Together, findings from these studies suggest that positivity is absent early in processing and emerges during more controlled stages of information processing.

Recent evidence based on neuroimaging also supports motivational accounts and speaks against neural degradation. Indeed, activation patterns in prefrontal regions associated with emotion regulation parallel the behavioral findings discussed above: Older versus younger adults recruit medial prefrontal regions (e.g., anterior cingulate) implicated in the regulation of emotion to a greater extent when processing negative versus positive images (Williams et al., 2006; Leclerc and Kensinger, 2011), suggesting that they actively down-regulate affective responses to negative but not positive stimuli. In a recent study by Ebner et al. (2012), older adults showed greater activation than younger adults in subregions of the dorsomedial prefrontal cortex (e.g., anterior cingulate and medial frontal gyrus) while processing angry versus happy faces.

Whereas prefrontal regions are recruited more for negative versus positive stimuli with age, activation in subcortical neural regions associated with emotional processing (e.g., amygdala) follows the opposite age-by-valence interaction (for a review, see Samanez-Larkin and Carstensen, 2011). In a seminal study by Mather et al. (2004), older adults showed greater amygdala activation while attending to and rating positive versus negative images, whereas amygdala activation in younger adults was equivalent across image valence. Recently Leclerc and Kensinger (2011) replicated the effect: younger adults showed greater amygdala activation in response to negative versus positive images. St. Jacques et al. (2010) posited that the distinct patterns of neural activation observed in prefrontal and subcortical regions are complementary. They proposed that increased motivation to regulate emotion leads older adults to actively engage the mPFC differently than younger adults, which in turn yields diverging amygdala activation patterns. Consistent with this interpretation, they found evidence of greater functional connectivity between the anterior cingulate cortex and right amygdala for older versus younger adults during the viewing and rating of emotionally salient images. In addition to attention and memory, positivity effects have been observed in neural regions involved in anticipatory reward. Whereas older and younger adults show similar levels of activation when anticipating rewards, only younger adults showed increased activation (caudate and insula) when anticipating losses (Samanez-Larkin et al., 2007).

Age differences in neural recruitment while processing positive versus negative information have also been observed at the level of whole-brain activity as indicated by late positive potential (LPP) brain waves (Kisley et al., 2007). The LPP waveform, which peaks several hundred milliseconds after stimulus onset (e.g., between 400 and 900 ms in the Kisley et al., 2007 study) tracks the relevance of stimuli (Schupp et al., 2000) and the allocation of attentional resources (Hajcak et al., 2006). Kisley et al. (2007) measured LPP within an adult sample while participants viewed and categorized a series of emotionally evocative images. Results indicated a systematic age-by-valence interaction in LPP amplitude consistent with the positivity effect: Whereas LPP amplitude did not differ by age in response to viewing positive images, the LPP amplitude evoked by negative images was inversely associated with age, indicating that older adults devote fewer neurocognitive resources to processing negative but not positive stimuli.

Reasoning from Cacioppo et al.’s (2011) aging-brain model, the positivity effect would emerge from dampened emotional responses to negative (but not positive) stimuli caused by selective neural degeneration in the amygdala. However, research findings reviewed above suggest that age differences appear in both negative *and* positive reactivity, and across subcortical *and* prefrontal regions. Given common brain regions for processing negative and positive stimuli, one would expect dampened reactivity to negative and positive stimuli. Moreover, the age-by-valence interactions in PFC activation suggest selective control of negative and positive. Specifically, older adults devote more neurocognitive resources to processing positive stimuli and down-regulating emotional responses to negative information. Thus, taken together, patterns of prefrontal and subcortical neural activity provide additional support for top-down processing.

## Malleable or Unreliable?

Several studies have not observed age differences in positivity, raising questions about the reliability and robustness of the effect (e.g., Kensinger et al., 2002; Grühn et al., 2005; Budson et al., 2006; Gallo et al., 2009). On close examination, however, the experimental designs in studies that fail to observe positivity also impose goals on participants that likely supplant chronically activated goals. Positivity is reduced when experimental instructions impose goals that interfere with chronically activated goals. That is, when experiments require participants to process stimuli in a particular way, e.g. by providing instructions about encoding stimulus valence (Kensinger et al., 2002) or asking participants to accurately remember all information (Grühn et al., 2005), positivity is not evident. In the latter study participants were asked to read lists containing positive, negative, and neutral words under the explicit instruction to “recall as many words as possible” for a subsequent memory test (Grühn et al., 2005, p. 582). Under these circumstances, both younger and older adults remembered more negative than positive words. An age-by-valence interaction was not observed.

On the other hand, positivity appears reliably when experiments do not impose constraints on processing; for example when participants are asked to simply “view” experimental materials, rather than explicitly process or commit them to memory. In such studies, positivity is observed in attention (e.g., Mather and Carstensen, 2003; Isaacowitz et al., 2006b) and memory (e.g., Charles et al., 2003; Kwon et al., 2009). We maintain that such approaches maximize the likelihood that chronically activated goals will influence cognitive processing. Our research group conducted two studies to explicitly test these contentions. The first study, by Löckenhoff and Carstensen (2007), found that when asked to simply review features of health care plans and physicians in order to choose among them, older adults disproportionately reviewed positive features of the alternatives. Positivity in review was eliminated, however, when experimental instructions explicitly primed informational goals (i.e., “please focus on specific facts and details”). Similar effects of goal manipulations on positivity were observed in autobiographical memory for emotional, mental, and physical well-being (Kennedy et al., 2004). In the latter study, the oldest (versus youngest) participants showed positive memory biases when their recall was prompted by open-ended instructions. However, when recall was prompted by instructions to focus on emotion or accuracy both age groups showed positive and negative memory biases, respectively^5^.

Although more research is needed, we expect that instructions that tell participants how to process information are likely to mask age differences in goals. These varied illustrations of the context sensitivity or malleability of the positivity effect support the theoretical contention that top-down processing is involved in positivity. Such findings also speak strongly against cognitive decline and neural degradation as the basis for positivity, because such explanations would not be sensitive to contextual cues.

## Adaptive or Maladaptive?

A key tenet of SST is that observed age differences in motivation reflect *adaptive* shifts in goals as people face changing time horizons. Generally speaking, maximizing information seeking and exploration is adaptive when time horizons are long whereas maximizing emotional well-being is adaptive when time horizons are relatively short. Of course, in the vast majority of studies on the positivity effect, there is no downside to attending to or remembering positive versus negative information in a biased manner. Fixating more on a happy versus angry face or remembering a photo of a smiling baby while forgetting one of a corpse has no detrimental consequences in the laboratory. But everyday life does present situations in which selective attention and memory are likely maladaptive. The question that arises is whether prioritization of positive information is set aside when stakes are high. Do older adults also show positive default processing tendencies when making high-stakes medical or financial decisions? That is, will positivity be observed when reviewing information about critical health care decisions (e.g., whether to treat cancer via surgery versus radiation therapy) and whether to invest retirement savings in a new company? Preliminary evidence suggests that the answer may be no. In a recent study by our research group (English, 2012), healthy and unhealthy older adults made a series of health-related (e.g., among physicians) and non-health-related decisions (e.g., among cars). Findings revealed significantly less positivity in health-related information review among participants in poor health relative to healthy participants. When making non-health-related decisions health status was unrelated to information review patterns. These findings indicate that older adults do indeed engage with negative material in contexts where avoiding it may have detrimental effects on well-being.

Older adults’ adaptive engagement with negative material also extends to situations involving threat. Prior research has found that younger people identify threatening (i.e., angry) faces faster than other emotions (for a review, see Vuilleumier, 2002), a pattern that has been interpreted as an adaptive and automatic response to threat (Öhman et al., 2001). Mather and Knight (2006) asked whether positivity in cognitive processing would preclude older people from displaying a similarly adaptive pattern. They administered a visual search task in which younger and older individuals were presented with an array of schematic faces containing eight neutral distractor faces and one target face depicting a happy, sad, or angry expression. Though theoretical accounts of positivity based on decline or degradation would predict age-related impairments in the speed of detecting angry versus happy faces, results indicated that older participants were faster to identify angry faces than happy or sad faces (younger adults showed a similar pattern). Thus, older adults prioritized the processing of negative over positive information when it held survival value (i.e., for angry but not sad faces).

Although evidence suggests that positivity is suppressed in situations where attending to negative information is adaptive, is positivity amplified when prioritizing emotional well-being is especially beneficial? There is some evidence that a positivity effect in gaze preferences is exacerbated in contexts that demand the regulation of emotion. Isaacowitz et al. (2008) observed minimal age differences in attentional preferences when individuals were induced into feeling neutral or positive moods^6^. However, when induced into negative moods, a robust positivity effect emerged. Younger adults oriented strongly toward negative faces whereas older adults oriented strongly toward positive faces. Thus, there is some intriguing evidence that older adults may actively deploy positivity to improve mood.

## Does the Positivity Effect Enhance or Impair Cognitive Processing?

One reasonable hypothesis, based on the literature reviewed above, is that older peoples’ preferential attention to and memory for positive versus negative information gives rise to suboptimal outcomes for decision making and deliberative problem solving. In this section we discuss empirical evidence that supports or contradicts such predictions.

As noted above, older adults disproportionately seek, attend to, and remember positive more than negative information when making decisions (Mather et al., 2005; Löckenhoff and Carstensen, 2007, 2008; Kim et al., 2008). But does this cause them to make poor choices? Extant research on risky and riskless decision making suggests that the answer, to date, is no. Mikels and Reed (2009) found that older adults were no more likely than younger adults to make suboptimal decisions (i.e., selecting an option with a lower expected value) when considering risky choices framed in terms of losses as opposed to gains. Another study using a sample of adults spanning the adult age range failed to observe age-by-valence interactions in risky choices; all age groups made objectively better decisions on gain versus loss trials (Weller et al., 2011). The positivity effect also does not appear to impair risk*less* decisions. Using multi-choice, multi-attribute decision tasks involving both positive and negative cues (i.e., choices among grocery stores and apartments), Hess et al. (2012) observed equivalent decision quality among younger and older adults. Evidence also suggests that the positivity effect does not impair – and may even benefit – subjective choice quality. For example, when older adults were asked to make lists of pros and cons to guide decisions among actual consumer products (i.e., a pen, mug, flashlight, and whiteboard) they reported more satisfaction than younger adults with their choices (e.g., Kim et al., 2008). By contrast, satisfaction did not differ across age groups when participants did not make pro-con lists prior to choosing. Taken together, the evidence thus far suggests that older adults’ preferential processing of positive versus negative information does not impair their decision making ability, and in some cases may lead to improved decision outcomes.

Given that effective interpersonal problem solving necessitates processing and acting upon negative and positive information, one might expect that older adults’ avoidance of negative information would be detrimental. Moreover, prior research points to an association between advanced age and the disproportionate use of avoidant versus instrumental strategies (e.g., Blanchard-Fields et al., 2007). Here too, however, evidence suggests that problem solving abilities improve with age. Despite older adults’ general preference for avoidant strategies, it appears that they apply a greater range of problem solving strategies more flexibly across situations compared to younger adults (for a review, see Blanchard-Fields, 2007).

By no means is the evidence on this point conclusive. Indeed, preferential processing of any category of stimuli is likely to involve some downside. However, in the domains of decision making and problem solving, findings to date fail to raise red flags.

## Future Directions

Although researchers have made a great deal of progress understanding the positivity effect, many questions remain. Evidence that positivity in cognitive processing is causally related to emotional well-being in older adults is scant, for example (for a discussion, see Isaacowitz and Blanchard-Fields, 2012). SST maintains that behavioral and cognitive selection operate in the service of emotion-related goals. Selective exposure is arguably the most effective way to regulate emotional states and there is considerable evidence that older people are more selective than younger people in their choice of social partners and environments (for a review, see Charles and Carstensen, 2010). Despite abundant evidence that older people are both relatively more selective (Charles and Carstensen, 2010) and experience a relatively positive balance of emotions in daily life (Carstensen et al., 2011), a causal link between selective exposure and emotional well-being has not been established.

Isaacowitz and Blanchard-Fields (2012) proposed the intriguing idea that positivity may also operate in the active regulation of negative mood states. To our knowledge, the mood-benefiting effects of positivity in online regulation have been demonstrated in only one study. Interestingly, findings suggested that executive control was a key moderator. Only older adults who had high levels of executive control and showed positive gaze preferences avoided negative mood changes (Isaacowitz et al., 2009b). This finding fits well with research conducted by Mather and others linking stronger evidence of positivity to greater cognitive control (e.g., Mather and Knight, 2005), and contributes to arguments that that top-down processing is required for deployment of goal-directed efforts.

Because laboratory experiments generally rely on weak emotional elicitors, such as synthetic face stimuli and word lists, which are unlikely to alter emotion states regardless of processing tendencies, strong tests of hypotheses about online regulation have yet to appear in the literature. Future research should examine the link between positivity in emotional processing and outcomes using stimuli that elicit stronger and more long-lasting effects on emotional experience.

Our research group has begun to test hypotheses about ways in which positivity may heighten older peoples’ susceptibility to problems encountered in everyday life, such as financial fraud. It is well-established that older people are the most frequent targets of financial scams and, for a variety of reasons, may be particularly susceptible, although great susceptibility has not been established (Shadel, 2012). Preferences that favor positive and ignore negative information could contribute to such susceptibility, either because potential warning signs are ignored or because messages about too-good-to-be-true prospects are especially salient. On the other hand, research reviewed above suggests that older people may discard positivity in high-stakes situations. Because of its dire consequences for older people, examining the role of positivity in fraud victimization is a worthwhile aim for future research.

Decision quality remains an important and understudied issue, and there are many reasons to expect that decision quality may suffer with age (see Peters et al., 2011, for an excellent review). Although, as noted above, recent findings offer no support for claims that positivity *per se* impairs older adults’ decision outcomes, the downstream effects of positivity in attention and memory on choice quality remain largely unexplored. To our knowledge no study has examined objective choice quality for actual (as opposed to hypothetical) decisions across age groups and as a function of information valence^7^. Given that millions of older adults are tasked with making important health-related decisions each year (e.g., Medicare Part D), it is imperative to understand whether motivations to seek positive and avoid negative information undermine the quality of these decisions. A related question for future research to consider is whether positive features of options drive choices more than negative features among older adults.

Both fraud victimization and decision making represent domains in which future research is needed to elucidate the precise conditions under which older adults’ relative bias toward the positive may be adaptive versus maladaptive. Such insights will have valuable applications to public policy: If, for example, older adults’ increased attention and memory for positive information improves their decision making for positively framed attributes, then physicians, hospitals, and policy makers might consider reframing decisions accordingly, so as to optimize choice quality.

The contributions of meaningfulness and time perspective to positivity have yet to be explored. SST maintains that age differences in the salience of emotionally meaningful goals are driven by constraints on future time. A considerable number of empirical studies in the realm of social choice support this contention (for a review, see Charles and Carstensen, 2010). To date, however, no studies have linked time horizons and meaningfulness to *positivity in cognitive processing*. SST predicts that relative to younger adults, older adults will devote more resources to processing highly meaningful information even if it engenders negative emotions. Although strong tests of this prediction have not been carried out, there is some suggestion that this may be the case. Fung et al. (2008) examined attentional preferences among Chinese residents of Hong Kong, a culture in which positive information is considered to be less meaningful than in Western cultures. Using the same eye-tracking paradigm as Isaacowitz et al. (2006b), Fung et al. found no evidence of positivity in the East Asian sample. In fact, older adults in their study demonstrated a greater preference for negative faces than did younger adults. Aside from illustrating that positivity may be culturally specific, these findings suggest the possibility that the positivity effect as it is typically observed does not depend on the valence of positive information *per se*, but rather the meaning attached to positive information. Because the study did not explicitly manipulate the meaningfulness of positive or negative stimuli, the lack of an observed positivity effect could reflect any number of cultural differences between Eastern and Western samples, of course, such as dialectical thinking or the degree to which mixed emotions are experienced. On the other hand, the positivity effect *has* been observed and replicated in picture memory among Korean samples (Kwon et al., 2009; Ko et al., 2011), adding further nuance to conclusions about cross-cultural relevance. To bring needed clarity to this area, future research would benefit from testing the positivity effect in contexts where emotional valence and meaningfulness can be better separated. In addition, because time horizons are the presumed theoretical drivers of age differences in goals that underlie the positivity effect, research on the role of perceived time in positivity is needed.

## Concluding Thoughts

As we have reviewed above, the motivational explanation for the positivity effect finds considerable support in the empirical literature. Research findings from dozens of studies are consistent with theoretically derived postulates that positivity reflects controlled cognition and chronically activated goals, is influenced by situational or contextual factors, and is largely adaptive for everyday functioning and well-being. Recent research has helped to illuminate conditions where the positivity effect is most and least likely to appear: Positivity appears when cognitive resources are available, when experimental tasks or stimuli do not activate automatic processing, and when information processing is unconstrained by external factors such as task instructions. In contrast, positivity is not observed when cognitive resources are significantly reduced (due to cognitive decline or experimental manipulations), when experimental tasks or stimuli elicit automatic processing or when situational demands supplant chronically activated goals. It appears increasingly that positivity may be reduced when the stakes are high. Taken together, the phenomenon appears to reflect a default cognitive processing approach in later life that favors information relevant to emotion-regulatory goals. Older people place high value on goals related to well-being and, all things being equal, cognitive processing operates under the influence of such goals.

In less than a decade, the positivity effect has become a well-replicated empirical observation. As more evidence accumulates and the guiding theory becomes more nuanced and detailed, it will be possible to test its various aspects with greater precision.

## Conflict of Interest Statement

The authors declare that the research was conducted in the absence of any commercial or financial relationships that could be construed as a potential conflict of interest.

## Footnotes

^1^Publication counts based on results of searching “positivity effect” in the PsychInfo database aging topic area.

^2^This is not to be confused with preferential memory and attention for emotional relative to neutral stimuli, which is relatively stable across age groups (see, for example, Murphy and Isaacowitz, 2008). Unfortunately, readers often mistake the headline conclusion from Murphy & Isaacowitz to mean that evidence for the positivity effect is qualified when in fact nearly three-quarters of the studies submitted to their meta-analysis lacked age comparisons of any kind and about half of the studies failed to include positive/negative contrasts. Only 15% of the studies Murphy & Isaacowitz included in their meta-analysis allowed for age and valence comparisons.

^3^It should be noted that the aging-brain model (Cacioppo et al., 2011) focuses solely on the downstream consequences of presumed amygdala dysfunction for memory and does not address the positivity effect in attention.

^4^Findings in this area are mixed. See Hot et al. (2012), in the current special issue for a review.

^5^Prior to providing retrospective reports, participants assigned to the accuracy-focused condition were told to “answer the questions as accurately as you can,” whereas participants in the emotion-focused condition were instructed to “focus on how you are feeling while answering the questions.” Participants in the control (i.e., open-ended) condition were simply asked to “answer the questions as you think you answered them back then.”

^6^In the neutral and positive affect conditions the only significant age difference was that younger adults attended more to positive faces compared to older adults.

^7^Although participants in the Kim et al. (2008) study made decisions about actual as opposed to hypothetical options, objective decision quality could not be examined because the options were roughly equivalent in value and utility.
